# Refining CFTR-Related Metabolic Syndrome (CRMS)/Cystic Fibrosis Screen Positive, Inconclusive Diagnosis (CFSPID) Diagnosis: Impact of CFTR2 Variant Classifications

**DOI:** 10.3390/ijns11030060

**Published:** 2025-07-30

**Authors:** MacKenzie Wyatt, Alexandra Quinn, Lincoln Shade, Meghan McGarry

**Affiliations:** 1 Department of Pediatrics, University of Washington School of Medicine, Seattle, WA 98105, USA; quinnalexandra66@gmail.com (A.Q.); meghan.mcgarry@seattlechildrens.org (M.M.); 2 Center for Respiratory Biology and Therapeutics, Seattle Children’s Research Institute, Seattle, WA 98101, USA; 3 Institute for Biomedical Informatics, Multidisciplinary Science, University of Kentucky, 725 Rose St Building, Suite 230, Lexington, KY 40536, USA; lincoln.shade@uky.edu

**Keywords:** cystic fibrosis, newborn screening, cystic fibrosis transmembrane conductance regulator-related metabolic syndrome, CRMS, CF screen positive inconclusive diagnosis, CFSPID, cystic fibrosis transmembrane conductance regulator gene, genetic testing, CRMS/CFSPID, sweat chloride

## Abstract

An unintended consequence of cystic fibrosis (CF) newborn screening (NBS) is the identification of infants with a positive NBS who do not meet the diagnostic criteria for CF (two CF-causing variants and/or sweat chloride > 60 mmol/L). This indeterminate diagnosis is called cystic fibrosis transmembrane conductance regulator (CFTR)-related metabolic syndrome (CRMS) or CF screen positive, inconclusive diagnosis (CFSPID). CRMS/CFSPID occurs when it is not clearly known whether *CFTR* variants are disease-causing. In 2024, the CFTR2 classification of many *CFTR* variants was changed from unknown significance to either CF-causing variants or variants of varying clinical consequences (VVCCs). We conducted a meta-analysis of CRMS/CFSPID cases from manuscripts to describe how the diagnoses would change using two different variant panels: (1) only CF-causing *CFTR* variants (Panel_CF-causing_) and (2) CF-causing variants and VVCCs (Panel_CF-causing+VVCCs_). Using the Panel_CF-causing_, 8.7% had two CF-causing variants (reclassified as CF), while 91.3% had less than two CF-causing variants (reclassified as Undetected). Using the Panel_CF-causing+VVCCs_, 51.4% had either two VVCCs or one VVCC with one CF-causing variant detected (reclassified as CRMS/CFSPD), 39.9% had less than two CF-causing variants detected (reclassified as Undetected), and 8.7% had two CF-causing variants (reclassified as CF). In conclusion, using the updated CFTR2 classification of *CFTR* variants significantly decreases the number of children with CRMS/CFSPID and gives a definitive diagnosis of CF to some children while not detecting as many children who are unlikely to develop CF.

## 1. Introduction

Cystic Fibrosis (CF) is an autosomal recessive genetic condition caused by variants in the *CFTR* gene, marked by disease in the lungs, pancreas, sinuses, and gastrointestinal tract [1]. Newborn screening (NBS) has been implemented in every state in the United States since 2010 [2]. CF NBS algorithms vary greatly within and between programs but in the US typically begin with an initial biomarker test, immunoreactive trypsinogen (IRT) [3]. Then, if IRT is elevated, the dried blood spot is tested for *CFTR* variants either in a panel or by genetic sequencing [4]. The next step is a sweat chloride test, which evaluates the concentration of chloride on the skin [5]. Sweat chloride is the gold standard diagnostic test for CF. One unintended consequence of CF NBS is the identification of infants who do not meet the diagnostic criteria for a formal CF diagnosis with a sweat chloride test (>60 mmol/L) [6]. Infants who have a positive newborn screen but do not meet the formal diagnostic criteria for CF can receive an indeterminate diagnosis known as cystic fibrosis transmembrane conductance regulator (*CFTR*)-related metabolic syndrome (CRMS) or CF screen positive, inconclusive diagnosis (CFSPID) [7]. Some *CFTR* variants (1) have an unknow impact on CF, or (2) cause CF only some of the time [8]. Uncertainty of *CFTR* variants leads to diagnosis of CRMS/CFSPID. Approximately 5–11% of children with CRMS/CFSPID develop clinical features of CF and convert to a CF diagnosis [9].

Not all variants in the *CFTR* gene definitively cause CF; some variants are benign, some are variants of varying clinical consequences (VVCCs), and the clinical impact of some variants is unknown [10]. The CFTR2 project is an international research project designed to improve the classification of the clinical impact of *CFTR* variants [11,12]. The CFTR2 database recently expanded the classification from 400 *CFTR* variants to over 1000 variants, with many variants of unknown significance reclassified as disease-causing.

Our study, a retrospective meta-analysis of CRMS/CFSPID, sought to understand the impact of the CFTR2 expansion of the classification of *CFTR* genetic variants on prior CRMS/CFSPID diagnoses from the literature. We hypothesized that children with CRMS/CFSPID would have more definitive reclassified diagnoses based on the updated classifications of *CFTR* genetic variants in the CFTR2 database.

## 2. Materials and Methods

The study population consisted of people diagnosed with CRMS/CFSPID and individual *CFTR* variants as reported in the literature. The primary endpoint was to determine the genetic diagnosis using the updated CFTR2 database [12]. We approached the task of reclassifying genetic diagnosis by utilizing (1) only the 1085 CF-causing variants (Panel_CF-causing_) and (2) the 1085 CF-causing variants and 55 VVCCs (Panel_CF-causing+VVCCs_). We included all studies that published the *CFTR* variants of children with a reported diagnosis of CRMS/CFSPID. We included studies that published the *CFTR* genetic variants for each individual and excluded any studies that published *CFTR* genetic variants by population variant frequency only. The inclusion criteria were articles of any cross-sectional, case-control, cohort, randomized controlled trial, as well as case reports. We excluded articles that were not available in English. This study was approved by the Institutional Review Board at Seattle Children’s Hospital.

### 2.1. Literature Search

**Figure 1:**
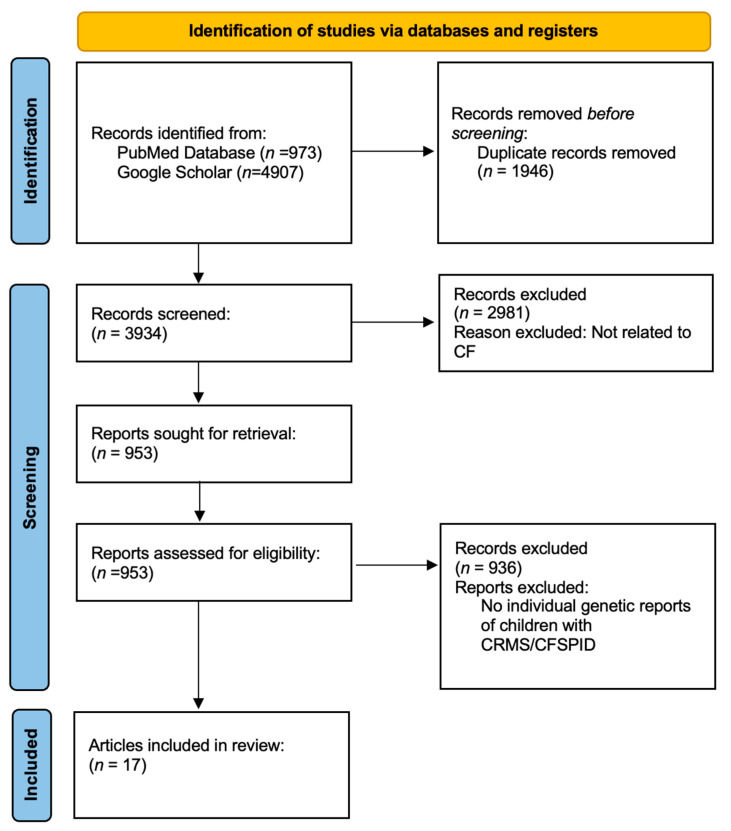
PRISMA flow diagram of literature review.

The authors followed the Preferred Reporting Items for Systematic Review and Meta-Analysis of Diagnostic Test Accuracy (PRISMA-DTA) [13]. The literature search started on 10 October 2024, and concluded on 15 February 2025. We searched for literature via PubMed and Google Scholar (Figure 1). The citations of identified manuscripts were searched for the relevant literature. The search keywords used were ‘CFTR-Related Metabolic Syndrome,’ ‘Cystic Fibrosis transmembrane conductance regulator related metabolic syndrome,’ ‘CRMS/CFSPID,’ ‘Cystic Fibrosis Screen Positive, Inconclusive Diagnosis,’ and ‘CFSPID.’ The search was conducted in English. All records were reviewed for inclusion of *CFTR* genetic variants. Two authors (MW and MM) conducted the initial search. The diagnostic criteria of CF and CRMS/CFSPID are shown in Table 1.

### 2.2. Data Extraction and Labeling

The literature and the Supplemental Data were extracted for *CFTR* variants, IRT, sweat chloride values, and age at diagnosis (conducted by MW and verified by MM and AQ). We gathered the pertinent data, including study identity (author and publication year), study characteristics (location, dates, study design, follow-up), and NBS information (NBS algorithm).

The literature was searched for duplicate entries of children with CRMS/CFSPID based on region, year of newborn screen, *CFTR* variants, IRT, sweat chloride concentration, and age of diagnosis. Duplicate entries of children from various studies were counted only once. Children with an initial elevated sweat chloride (>60 mmol/L) were excluded, since they met the diagnosis of CF.

The clinical interpretation of *CFTR* variants was determined based on the CFTR2 database (conducted by MW and verified by MM). If the *CFTR* variant was in the CFTR2 database, it was classified as either ‘CF-causing,’ ‘variant of varying clinical consequences (VVCC),’ or ‘non-CF-causing.’ If a *CFTR* variant was not in the database, it was labeled ‘variant of unknown significance (VUS).’ If a child did not have a *CFTR* variant found, it was labeled as ‘not identified.’ Variant category definitions are given in Table 2.

### 2.3. Diagnostic Criteria for Reclassified Diagnosis Based on CFTR2 Database

We reclassified the reported diagnoses based on the updated *CFTR* genetic variant classifications in the CFTR2 database. Based on a child’s genetic variants’ clinical implications, we determined their genetic diagnosis (Table 3) by two mechanisms. First, using Panel_CF-causing_, reclassification diagnosis was determined as ‘CF’ if a child had 2 CF-causing variants. Otherwise, a child was reclassified as ‘Undetected’ for any other combination of variants. Second, using Panel_CF-causing+VVCCs_, reclassification diagnosis was determined as CRMS/CFSPID if there were two *CFTR* variants (CF-causing and 1 VVCC or 2 VVCCs), ‘CF’ if a child had two CF-causing variants, or ‘Undetected’ if there were fewer than two CF-causing or VVCC *CFTR* variants detected.

### 2.4. Sub-Analysis of Children with CRMS/CFSPID Who Converted

All the children included in our meta-analysis had been clinically diagnosed with CRMS/CFSPID, which we will refer to as the ‘reported diagnosis’. Some manuscripts reported data on children with CRMS/CFSPID who converted to CF (CRMS/CFSPID→CF) or CFTR-related disorder (CRMS/CFSPID→CFTR-RD). We performed a sub-analysis on these children.

### 2.5. Secondary Outcomes Analyses

We compared the initial sweat chloride concentration and IRT level between the reclassified diagnoses using Panel_CF-causing+VVCCs_.

### 2.6. Statistical Analyses

Descriptive statistics are presented as median (interquartile range [IQR]) for continuous variables and number (%) for categorical variables. For initial group comparisons, we used Mann–Whitney U, Cochran’s Q statistic, Stuart–Maxwell, Wilcoxon signed-rank, chi-squared, or McNemar tests, as appropriate. Statistical significance was defined as *p*-value < 0.05. Statistical analyses were performed using R version 4.0.1.1. Figures were created using the R ggplot2 package. ChatGPT 4.o was used only to revise the coding for statistical analysis.

## 3. Results

### 3.1. Literature Review

Upon searching PubMed, Google Scholar, and the citations of identified manuscripts, 17 papers met the inclusion criteria (Figure 1). Of these 17 manuscripts, nine were from Europe, five were from North America, one was from Australia, and two were from North America and Europe (Table 4). After reviewing the manuscripts, 537 children with CRMS/CFSPID were identified. We excluded two children for an initial sweat chloride >60 mmol/L. We excluded duplicative data from 19 children who had data in multiple manuscripts in our study and only counted them once. There were duplicates of twelve children in the papers from California, USA, and duplicates of seven children in the papers from Italy that were excluded. A total of 516 children with CRMS/CFSPID were included for analysis. Of the 516 children with CRMS/CFSPID, 136 children had a reported diagnosis of CRMS/CFSPID→CF and 15 children had a reported diagnosis of CRMS/CFSPID→CFTR-RD.

### 3.2. Primary Outcomes: Reclassification Diagnoses

**Figure 2:**
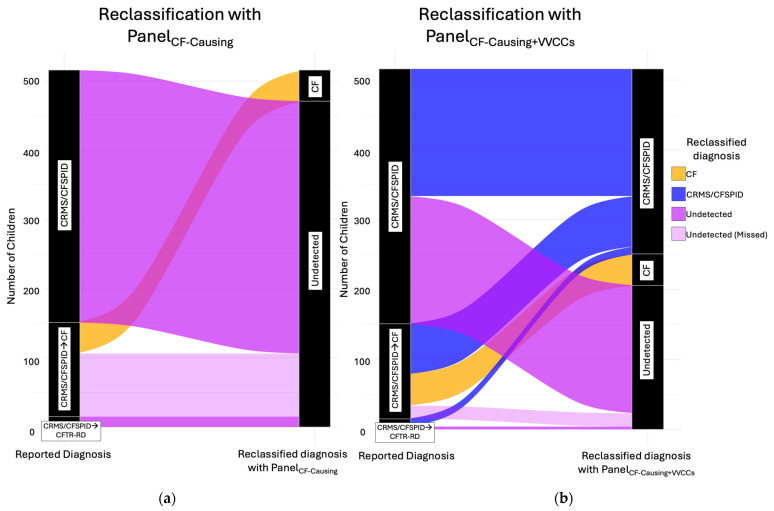
Reclassification Diagnoses of CRMS/CFSPID cases reported in the literature using 2 Variant Panels: (**a**) reclassification diagnosis using Panel_CF-causing_ eliminates CRMS/CFSPID diagnoses; (**b**) reclassification diagnosis using Panel_CF-causing+VVCCs_ retains more children with CRMS/CFSPID and misses less children who convert to CF.

When using Panel_CF-causing_, a child could be reclassified with the diagnosis of ‘CF’ or ‘undetected.’ There were no genetic diagnoses of ‘CRMS/CFSPID’ and thus the diagnosis was eliminated. In comparison, when using a CF-causing and VVCC variant panel, children were reclassified with ‘CF,’ ‘CRMS/CFSPID,’ or ‘undetected.’ (Table 5, Figure 2).

First, using Panel_CF-causing_, 45 (8.7%) children would have two CF-causing variants, and their reclassified diagnosis was CF. Otherwise, 471 (91.3%) children would have fewer than two CF-causing variants identified, and their reclassified diagnosis was undetected. No children would have a reclassified diagnosis of CRMS/CFSPID.

Next, using Panel_CF-causing+VVCCs_, 264 (51.1%) children would have two *CFTR* variants detected (1 CF-causing and 1 VVCC or 2 VVCCs), and their reclassified diagnosis was CRMS/CFSPID. Using Panel_CF-causing+VVCCs,_ 45 (8.7%) children would have a reclassified diagnosis of CF, which is the same percentage as with the Panel_CF-causing_. Otherwise, using Panel_CF-causing+VVCCs,_ 207 (40.0%) children would have less than two variants identified, and their reclassified diagnosis was undetected.

Four children had complex variant combinations (more than two variants identified): CF-causing variant/VVCC and VVCC; CF-causing variant/VVCC, VVCC, and VVCC; CF-causing variant and VVCC/non-CF-causing variant; VVCC, VVCC, and VVCC/VVCC and VVCC.

There was substantial heterogeneity for the presence of one CF-causing variant within the studies (*I*^2^ = 72.4%) (Figure S1). There was considerable heterogeneity for the presence of two CF-causing variants within the studies (*I*^2^ = 77.4%) (Figure S2).

### 3.3. Subanalysis of Children with CRMS/CFSPID Who Converted

The *CFTR* genetic variants of 136 children with CRMS/CFSPID→CF are shown in Table 6. First, using Panel_CF-causing_, 91 (66.9%) children with a reported diagnosis of CRMS/CFSPID→CF would be ‘undetected’ and thus missed for CF diagnosis. Second, using Panel_CF-causing+VVCCs_, 71 (52.2%) children with CRMS/CFSPID→CF have a reclassified diagnosis of CRMS/CFSPID, and only 19 (14.0%) children with CRMS/CSPID→CF would be ‘undetected’ and thus missed for CF diagnosis (Figure 2).

More children remain classified as CRMS/CFSPID, and fewer children with a reported diagnosis of CRMS/CFSPID→CF would be missed with the Panel_CF-causing+VVCCs_ compared to using only using Panel_CF-causing_ (Stuart-Maxwell *p*-value < 0.05).

### 3.4. Secondary Outcomes: IRT/Sweat Chloride Data

**Figure 3:**
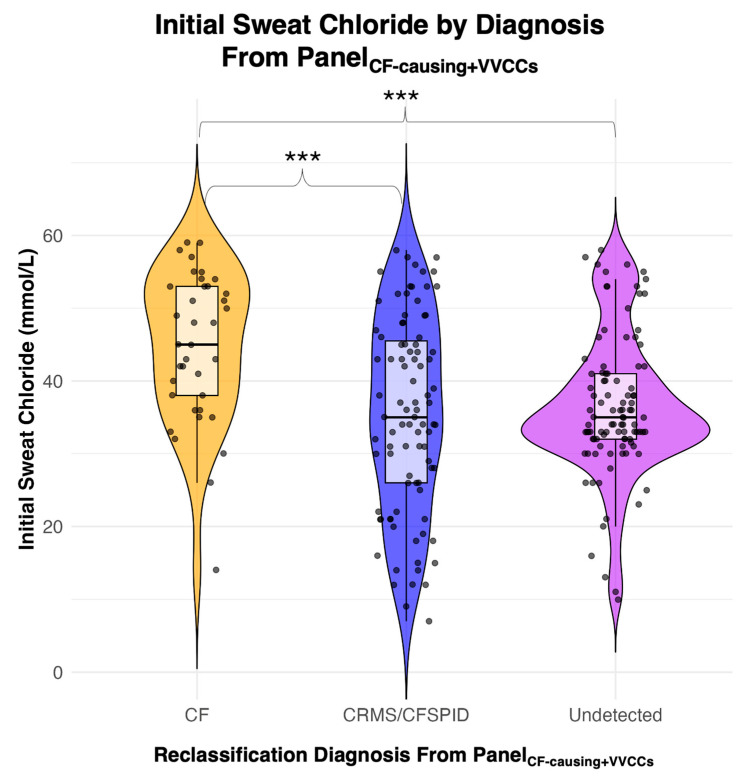
Initial sweat chloride by reclassification diagnosis from Panel_CF-causing+VVCCs_. *** Statistical significance *p*-value < 0.05.

Initial sweat chloride concentration data were available for 227 children. The mean initial sweat chloride concentration level for all children with CRMS/CFSPID was 37.3 mmol/L (95%CI 25.5–49.1). The mean sweat chloride concentration level was statistically significantly higher in the 37 children reclassified from Panel_CF-causing+VVCCs_ as CF (44.7, 95% CI 34.4–50.0) compared to the 91 children reclassified as CRMS/CFSPID (35.4, 95% CI: 22.1–48.7) and the 99 children reclassified as undetected (36.4, 95% CI: 26.7–46.1, Kruskal–Wallis *p* < 0.001) (Figure 3).

Initial IRT data were available for 75 children. The mean initial IRT level for all children with CRMS/CFSPID was 107.0 ng/mL (95%CI 43.18–170.8). Using Panel_CF-causing+VVCCs_, initial IRT levels did not differ significantly between children reclassified as CF, CRMS/CFSPID, or undetected (Kruskal–Wallis *p* = 0.15) (Supplemental Information).

## 4. Discussion

In this meta-analysis of published cases of CRMS/CFPSID, we found that using *CFTR* genetic classifications based on the 2024 CFTR2 database improves definitive diagnoses in children previously diagnosed with CRMS/CFSPID. Using a Panel_CF-causing,_ one could eliminate CRMS/CFSPID diagnoses by genetic classification. However, using a Panel_CF-causing_ would miss 91 (66.9%) of published CRMS/CFSPID→CF conversions. These cases would be a false negative newborn screen and would need to be diagnosed based on clinical symptoms, thus leading to delayed CF diagnoses. Using Panel_CF-causing+VVCCs,_ CRMS/CFPSID diagnoses would be reduced but not eliminated. Panel_CF-causing+VVCCs_ would detect a higher percentage of those with CRMS/CFSPID conversions but still miss 19 (14.0%) of the published CRMS/CFSPID→CF conversions.

There is still controversy over detecting CRMS/CFPSID by newborn screening [31]. Some people believe CRMS/CFSPID should not be detected by newborn screening for multiple reasons. Being diagnosed with CRMS/CFSPID can lead to anxiety for families due to the uncertainty and can lead to unnecessary overmedicalization and testing [32,33,34,35,36,37,38,39]. There was no standardized guidance on management for these children until 2024, so it has been confusing for providers on the follow-up of these children [40]. Some newborn screening programs detect more CRMS/CFSPID than CF [31]. Additionally, CRMS/CFSPID can lead to valuable resources and staffing needs for CF centers [41]. These resources can take away from children with CF. These children must be followed by CF centers, and thus appointment slots for sweat testing and physician follow-up are being utilized. There is a low conversion rate of CRMS/CFSPID→CF, so some programs do not feel as though they need to be detected via newborn screening and should be diagnosed later in life [21].

Other programs believe CRMS/CFSPID should be detected by newborn screening. Some centers’ goals are to identify every child who is at risk for converting to CF and CFTR-RD. Some centers have the resources to follow all these children. It is important to consider that children of minoritized races, ethnicities, or ancestries face increased barriers to being diagnosed after a false negative newborn screening, as there is still a false perception that CF occurs in a white, European population, despite CF occurring in all populations across the world [42,43].

Using both strategies with CFTR2 (only CF-causing variants or CF-causing variants and VVCCs) as part of a newborn screen leads to faster definitive diagnoses for children and less uncertainty, as more children are initially classified as CF or undetected, and fewer children are initially classified as CRMS/CFSPID. Using only CF-causing variants eliminates the diagnosis of CRMS/CFSPID, but more children with a reported diagnosis of CRMS/CFSPID→CF are undetected and thus missed. Using CF-causing variants and VVCCs still retains the diagnosis of CRMS/CFSPID, but it reduces the number of children who have this label and leads to more children with CRMS/CFSPID→CF who retain the diagnosis of CRMS/CFSPID to be followed for diagnostic conversion. Children with CRMS/CFSPID largely fall into two categories: those who will convert to CF and those who have a persistent CRMS/CFSPID diagnosis [44]. By applying the new knowledge we have with the CFTR2 database, we reduce the number of children with CRMS/CFSPID and improve the specificity of the diagnosis of CRMS/CFSPID. Our meta-analysis is one of the most extensive studies of children with CRMS/CFSPID and their individual *CFTR* variant combinations and helps illuminate the impact of the CFTR2 database on diagnoses. Our findings are similar to past studies that show that a child with a CF-causing variant and a VVCC is more likely to convert from CRMS/CFSPID to CF [45].

We found that initial sweat chloride levels were significantly higher in children who were reclassified as CF compared to CRMS/CFSPID or undetected. A sweat chloride test is the gold standard diagnostic test for CF, so these findings support diagnosis by genetic reclassification based on the CFTR2 database. Prior studies have examined the relationship between the increase in sweat chloride concentration over time and CRMS/CFSPID→CF diagnosis. Salinas et al. (2023) showed that a gradual increase in sweat chloride is associated with an increased risk of converting from CRMS/CFSPID to CF, but Terlizzi et al. (2023) showed more variability in the sweat chloride [22,46]. The IRT was not significantly different between groups, which differs from some of the literature that suggests a higher IRT predicts conversion from CRMS/CFSPID→CF [45]. Some studies have found that IRT concentration is higher in CRMS/CFSPID→CF compared to CRMS/CFSPID [24]. There has not been an explicit cutoff for IRT that is predictive of a child with CRMS/CFSPID→CF [16,47].

These findings are important in light of the 2025 CF Foundation CF newborn screening guidelines, which allow newborn screening programs to choose to perform sweat testing on children with only CF-causing variants or CF-causing variants and VVCCs, which will impact CRMS/CFSPID diagnoses [2]. Genetic sequencing labs can allow NBS programs to determine whether the results will reveal only the CF-causing variants or the CF-causing variants and VVCCs [48]. Using only CF-causing variants decreases the number of sweat tests that a center must perform. Some CF centers do not have the staffing to conduct all the sweat tests in a reasonable time, which can delay CF diagnoses. Additionally, using a panel of only CF-causing variants eliminates the diagnosis of CRMS/CFSPID. The only way a child would have CRMS/CFSPID would be if the algorithm had a very high IRT cutoff. Using only either panel and calling out only those with two variants eliminates the number of children a CF center must follow, as some centers do not have enough CF providers and appointment slots. The downside of using only CF-causing variants is that a significant number of children with CRMS/CFSPID→CF would be reclassified as undetected unless they had a very high IRT level and thus would not be followed. This can delay diagnosis and treatment for children with CRMS/CFSPID→CF, which has been shown to lead to worse clinical outcomes [43].

Using CF-causing variants and VVCCs in newborn screens and diagnostic testing is beneficial as it can reduce the amount of CRMS/CFSPID while also retaining a smaller group of children with CRMS/CFSPID to follow. This increases the likelihood that a child will convert from CRMS/CFSPID→CF within this group, making the diagnosis more specific and useful. Using this method increases the number of children requiring a sweat chloride concentration and to be followed by a CF center. Not only does this lead to increased staffing and resource needs at the CF center, but sweat chloride concentrations and clinic visits are expensive and time-consuming for families as well. Using VVCCs can be important for health equity. Although reported race and ethnicity is a social construct and a poor proxy of genetic ancestries, some variants are rarer overall but occur more frequently in racial and ethnic minority groups [42,49]. This is a problem within CF, and any genetic disease where variant panels were developed based on a predominantly non-Hispanic white or European populations [50]. This bias leads to misdiagnoses and delayed care [43].

When choosing which NBS algorithm and variant panel to use, the newborn screening program must determine its goals. Some programs want to detect every child with CF or at risk of CF, regardless of the number of children with an indeterminate diagnosis. Other programs have the goal of establishing a definitive diagnosis for families of CF and minimizing the number of those with CRMS/CFSPID. We have shown that using a variant panel with only CF-causing variants or CF-causing variants and VVCCs, the amount of CRMS/CFSPID is reduced as more children will be definitively diagnosed with CF sooner, and fewer healthy children will receive a CRMS/CFSPID diagnosis. We have shown that a variant panel with VVCCs detects more children who convert from CRMS/CFSPID→CF compared to a variant panel with only CF-causing variants and leads to fewer missed cases.

This study highlights the importance of investigating to ascertain increased knowledge of *CFTR* variants. It is important to understand which VVCCs are more likely to be CF-causing vs normal, and which VVCCs in combination with certain variants are more likely to be CF-causing. CF centers should be reviewing their cases from their center, paying attention to the variants and any false negative NBS cases to ensure their methods are capturing their population. Additionally, they should be reviewing their cases to ensure equity. As populations evolve to become more diverse, missed cases may become more prevalent in areas that previously were more homogenous [42]. Public health measures should be continually reassessed and modified to ensure equity. Our study demonstrates the importance of publishing newborn screening data to help further this knowledge of the clinical impact of *CFTR* variants. Most newborn screening programs did not have any representation in the literature, whereas some centers in Italy and Canada are very well represented. This study was made possible by prior publications and the Supplemental Data. Our study demonstrates the need to understand how other biomarkers and clinical factors predict CRMS/CFSPID conversion to CF [45]. Terlizzi et al. show that the literature on CRMS/CFSPID is varied and highlights the need for an international registry for CRMS/CFSPID [45].

Although our findings were specific to CF, the same principles can be applied to any newborn screen that has a genetic component. Newborn screens that include genetic testing can have these indeterminate results as well, including Severe Combined Immunodeficiency (SCID), metabolic disorders, and congenital hypothyroidism [51,52,53]. The American College of Medical Genetics and Genomics has categories of variant classification that include variants of varying clinical consequences with different nomenclature [54]. They categorize genes as likely benign or likely pathogenic when we know the certainty of its influence on disease (at least 90%) or a variant of unknown significance when the certainty is less than 90% [54]. These genes can also be reclassified as benign or pathogenic as more information becomes known. Variants in classic galactosemia, phenylketonuria, and medium-chain acyl-CoA dehydrogenase (MCAD) deficiency have all had variants reclassified as more information becomes available [55]. Some children have variants that must be followed to ensure no symptoms at a later date, which leads to a similar pattern. Our study highlights the need to pool resources and gather information to understand variants to obtain more definitive diagnoses.

One limitation of our study is reporting bias. Few NBS programs publish any data on their CRMS/CFSPID newborn screening. There is likely a preference to publish cases of children with CRMS/CFSPID who convert to CF over cases of just CRMS/CFSPID. Thus, we could be overrepresenting the children with CRMS/CFSPID who convert to CF. Another limitation of this study is that there are differing durations of follow-up for each study, and theoretically, children can convert from CRMS/CFSPID to CF or CFTR-RD at any given time. Some children likely converted to CF after publication, so the reported diagnosis could be underrepresenting CRMS/CFSPID→CF and can thus be underrepresenting the amount of missed diagnoses from the proposed strategies. Another limitation is the heterogeneity of algorithmic differences based on geography. Some children may be diagnosed with CRMS/CFSPID in one location and followed at another location where they converted to CF and thus would not be documented. A child with the same variants would be more likely to be diagnosed with CRMS/CFSPID in a location that screens for more *CFTR* variants. Despite these limitations, our findings are valid and important for modeling how CFTR2 could impact diagnosis. It provides an example for CF centers to use when analyzing their own prior data to decide how to proceed with variant panels or genetic sequencing as more states and regions begin to adopt the recommendations from the new newborn screening guidelines.

Our study has a diversity of children from a variety of areas around the world that have differing NBS algorithms. Additionally, we applied the same diagnostic reclassification schema based on CFTR2, which increases generalizability. This strengthens the generalizability of our study. Our generalizability is limited because each study followed children for differing amounts of time. Our study does have high heterogeneity (see Supplemental Data), as each region has differing NBS algorithms, but CFTR2 is sourced from all over the world and is very robust, strengthening our findings [12].

## 5. Conclusions

The expansion of CFTR2 improves definitive diagnosis of CF and reduces the number of children with uncertain diagnoses with CRMS/CFSPID. We show that using only CF-causing variants removes CRMS/CFSPID cases but misses more children who convert from CRMS/CFSPID→CF compared to using CF-causing variants and VVCCs.

## Figures and Tables

**Table 1:** Diagnostic criteria for CF and CRMS/CFSPID [6].

Diagnostic Criteria for CF and CRMS				
Genetic CFTR variants		Sweat chloride		
Variant 1	Variant 2	Normal (<30 mmol/L)	Intermediate (30–60 mmol/L)	Elevated (>60 mmol/L)
CF-causing	CF-causing	CF	CF	CF
CF-causing, VVCC, or VUS	VVCC or VUS	CRMS	CRMS	CF
CF-causing, VVCC, or VUS	Non CF-causing	Normal (Carrier)	CRMS	CF
Non CF-causing	Non CF-causing	Normal	CRMS	CF

**Table 2:** Definitions of variant categories used in this paper.

Variant Interpretation Terms	Definition
CF-causing variant	A *CFTR* variant that causes CF when in *trans* with another CF-causing variant
Variant of varying clinical consequences (VVCC)	A *CFTR* variant that causes CF in some children but not others when in *trans* with another CF-causing variant.
Non CF-causing	A *CFTR* variant that does not cause CF.
Variant of unknown significance (VUS)	A *CFTR* variant that is not described in the CFTR2 database.
Not identified	No *CFTR* variant identified.

**Table 3:** *CFTR* variant reclassification diagnostic schema.

	*CFTR* Variant Reclassification Diagnostic Schema					
		Variant 1				
		CF-causing	VVCC	Non CF-causing	VUS	Not identified
Variant 2	CF-causing	CF	CRMS/CFSPID or Undetected ***	Undetected	Undetected	Undetected
	VVCC		CRMS/CFSPID or Undetected ***	Undetected	Undetected	Undetected
	Non CF-causing			Undetected	Undetected	Undetected
	VUS				Undetected	Undetected
	Not identified					Undetected

*** Undetected using Panel_CF-causing_; CRMS/CFSPID using Panel_CF-causing+VVCCs_.

**Table 4:** Manuscripts included in meta-analysis.

1st Author, Year	Location	Study Type	Years of Study	Total CRMS	CRMS→CF	CRMS→CFTR-RD	IRT	Initial Sweat Chloride
Castaldo A, 2020 [14]	Italy	Retrospective cohort	2008–2019	99	2	8	✔	✔
Ooi CY, 2015 [15]	Canada, Italy	Prospective cohort	2007–2013	82	9			✔
Munck A, 2020 [16]	France	Prospective cohort	2002–2009	70	21			✔
Gunnett MA, 2023 [17]	USA	Retrospective cross-sectional	2008–2020	63	11			✔
Terlizzi, Vito, 2019 [18]	Italy	Retrospective cross-sectional	2011–2016	50	5			✔
Hatton A, 2022 [19]	Poland	Case series	2006–2016	23	4			✔
Rock MJ, 2023 [20]	USA	Retrospective cross-sectional	2016–2021	22				✔
Terlizzi V, 2021 [21]	Italy	Prospective cohort	2011–2018	22	18	4		✔
Salinas DB, 2023 [22]	USA	Retrospective cross-sectional	Before 2023	20	12			
Kharrazi M, 2015 [23]	USA	Cross-sectional	2007–2012	20	20			
Ooi CY, 2019 [24]	Canada, Italy	Prospective cohort	2007–2016	14	14		✔	✔
Groves T, 2015 [25]	Australia	Retrospective cohort	1996–2010	14	14		✔	✔
Çoksüer F, 2025 [26]	Turkey	Retrospective cohort	2015–2023	11			✔	✔
Terlizzi V, 2021 [27]	Italy	Prospective cohort	2018–2020	11	2		✔	✔
Dolce D, 2023 [28]	Italy	Retrospective cohort	2011–2018	10	7	3		✔
Ginsburg D, 2022 [29]	USA	Case series	Before 2022	10	10		✔	✔
Skov M, 2020 [30]	Denmark	Retrospective cross-sectional	2016–2018	3			✔	✔

**Table 5:** Reclassification diagnostic schema of all children with CRMS/CFSPID.

	*CFTR* Variant Types of Children with CRMS/CFSPID					
		Variant 1				
	(*n* = 516 *)	CF-causing	VVCC	Non CF-causing	VUS	Not identified
Variant 2	CF-causing	45 (8.7%)	243 (47.1%)	50 (9.7%)	47 (9.1%)	45 (8.7%)
	VVCC		21 (4.1%)	15 (2.9%)	10 (1.9%)	8 (1.6%)
	Non CF-causing			2 (0.4%)	2 (0.4%)	2 (0.4%)
	VUS				1 (0.2%)	1 (0.2%)
	Not identified					23 (4.5%)

* One child had a complex variant combination and was not included in a single category: CF-causing variant + VVCC in cis with 1 non CF-causing variant.

**Table 6:** Reclassification diagnostic schema of children with CRMS/CFSPID→CF.

	*CFTR* Variant Types of Children with CRMS/CFSPID→CF					
		Variant 1				
	(*n* = 136 *)	CF-causing	VVCC	Non CF-causing	VUS	Not identified
Variant 2	CF-causing	45 (33.1%)	68 (50%)	2 (1.5%)	8 (5.9%)	8 (5.9%)
	VVCC		3 (2.2%)	---	1 (0.7%)	---
	Non CF-causing			---	---	---
	VUS				---	---
	Not identified					---

* One child had a complex variant combination and was not included in a single category: CF-causing variant + VVCC in cis with 1 non CF-causing variant.

## Data Availability

All participant data that underlie the results reported in this manuscript are publicly available and deidentified. Individual participant data that underlie the results reported in this manuscript and a corresponding data dictionary will be shared electronically with other researchers for the purpose of conducting systematic reviews with meta-analyses and upon approval by the corresponding author. For this, researchers requesting the data will need to have their study approved by an independent review committee (such as an institutional review board) and directly contact the corresponding author.

## Supplementary Materials

The following supporting information can be downloaded at: https://www.mdpi.com/article/10.3390/ijns11030060/s1, Figure S1: Forest plot of 1 CF-causing variant; Figure S2: Forest plot of 2 CF-causing variants.

## Abbreviations

The following abbreviations are used in this manuscript:

CF	cystic fibrosis
NBS	newborn screening
CFTR	cystic fibrosis transmembrane conductance regulator
CRMS	cystic fibrosis transmembrane conductance regulator (CFTR)-related metabolic syndrome
CFSPID	cystic fibrosis screen positive, inconclusive diagnosis
CFTR2	The Clinical and Functional TRanslation of CFTR
IRT	immunoreactive trypsinogen
VVCCs	variant of varying clinical consequences
PRISMA-DTA	Preferred Reporting Items for Systematic Review and Meta-Analysis of Diagnostic Test Accuracy
VUS	variant of unknown significance
CFTR-RD	CFTR-related disorder
Panel_CF-causing_	panel using only CF-causing variants
Panel_CF-causing+VVCCs_	panel using CF-causing variants and VVCCs
IQR	interquartile range
PKU	phenylketonuria
MCAD	medium-chain acyl-CoA dehydrogenase
