# Supplementary figures and images for: Refining CFTR-Related Metabolic Syndrome (CRMS)/Cystic Fibrosis Screen Positive, Inconclusive Diagnosis (CFSPID) Diagnosis: Impact of CFTR2 Variant Classifications
Source: Int J Neonatal Screen. 2025 Jul 30;11(3):60. doi: 10.3390/ijns11030060 (PMC12372008; doi:10.3390/ijns11030060)

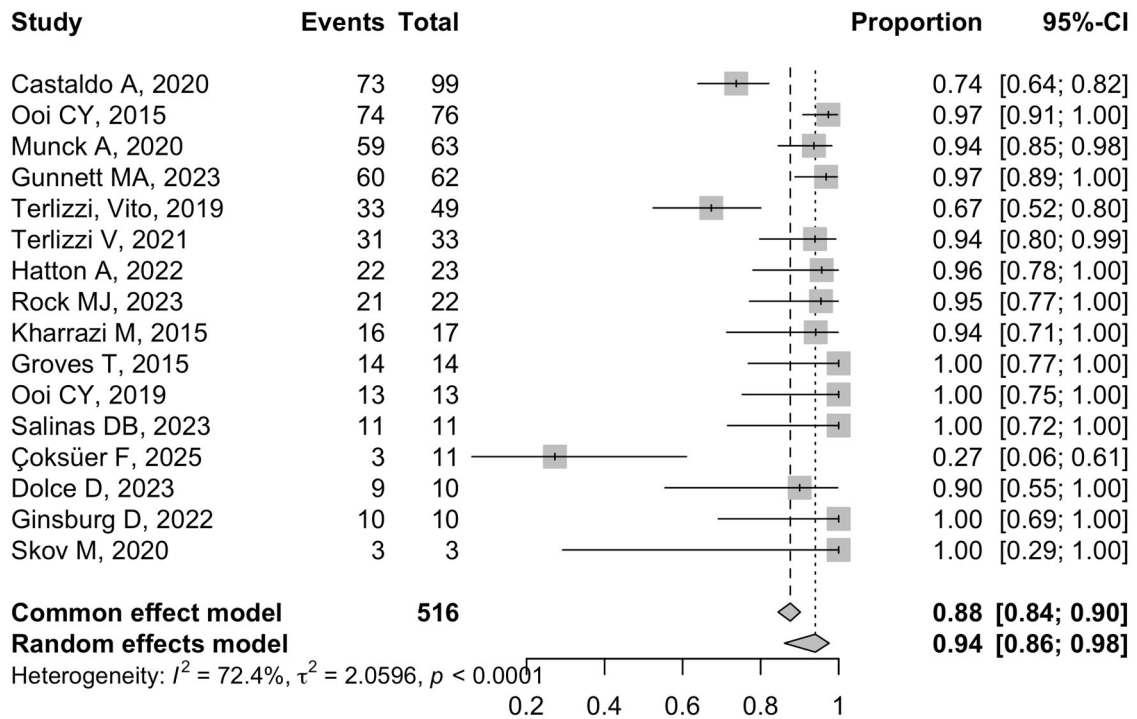

Figure S1. Forest plot of 1 CF-causing variant.

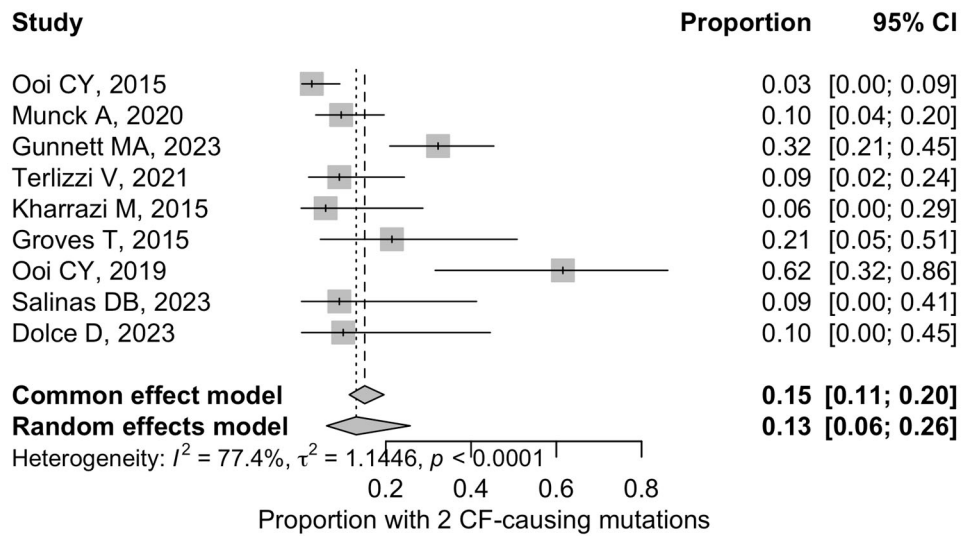

Figure S2. Forest plot of 2 CF-causing variants.

Supplement: Supplementary file 1 [file IJNS-11-00060-s001.zip › IJNS-3733604-supplementary.pdf]
